# Comprehensive screening shows that mutations in the known syndromic genes are rare in infants presenting with hyperinsulinaemic hypoglycaemia

**DOI:** 10.1111/cen.13841

**Published:** 2018-09-20

**Authors:** Thomas W. Laver, Matthew N. Wakeling, Janet Hong Yeow Hua, Jayne A. L. Houghton, Khalid Hussain, Sian Ellard, Sarah E. Flanagan

**Affiliations:** ^1^ Institute of Biomedical and Clinical Science University of Exeter Medical School Exeter UK; ^2^ Paediatric Department Hospital Putrajaya Putrajaya Malaysia; ^3^ Department of Molecular Genetics Royal Devon and Exeter NHS Foundation Trust Exeter UK; ^4^ Department of Pediatric Medicine Division of Endocrinology Sidra Medicine Doha Qatar

**Keywords:** genetic screening, hyperinsulinaemia hypoglycaemia of infancy, medical genetics, molecular diagnostics, mutation, neonatal hyperinsulinism, syndrome

## Abstract

**Objective:**

Hyperinsulinaemic hypoglycaemia (HH) can occur in isolation or more rarely feature as part of a syndrome. Screening for mutations in the “syndromic” HH genes is guided by phenotype with genetic testing used to confirm the clinical diagnosis. As HH can be the presenting feature of a syndrome, it is possible that mutations will be missed as these genes are not routinely screened in all newly diagnosed individuals. We investigated the frequency of pathogenic variants in syndromic genes in infants with HH who had not been clinically diagnosed with a syndromic disorder at referral for genetic testing.

**Design:**

We used genome sequencing data to assess the prevalence of mutations in syndromic HH genes in an international cohort of patients with HH of unknown genetic cause.

**Patients:**

We undertook genome sequencing in 82 infants with HH without a clinical diagnosis of a known syndrome at referral for genetic testing.

**Measurements:**

Within this cohort, we searched for the genetic aetiologies causing 20 different syndromes where HH had been reported as a feature.

**Results:**

We identified a pathogenic *KMT2D* variant in a patient with HH diagnosed at birth, confirming a genetic diagnosis of Kabuki syndrome. Clinical data received following the identification of the mutation highlighted additional features consistent with the genetic diagnosis. Pathogenic variants were not identified in the remainder of the cohort.

**Conclusions:**

Pathogenic variants in the syndromic HH genes are rare; thus, routine testing of these genes by molecular genetics laboratories is unlikely to be justified in patients without syndromic phenotypes.

## 1 INTRODUCTION

Congenital hyperinsulinaemic hypoglycaemia (HH) is a disorder where episodes of hypoglycaemia are caused by unregulated insulin secretion despite low blood glucose levels. Prompt treatment is crucial in order to avoid serious lifelong complications such as seizures and permanent brain injury.^1^ Genetic testing is important for the clinical management of this condition as identifying the underlying genetic aetiology will inform on the pancreatic histology which, for patients who are unresponsive to medical treatment, will help to determine whether a lesionectomy or a near‐total pancreatectomy is required.^2^

HH is most commonly the result of a monogenic aetiology with mutations in at least eight genes reported to cause isolated disease.^3^ Routine screening of these genes using a combination of rapid Sanger sequencing and targeted next‐generation sequencing identifies a mutation in approximately 40%‐50% of cases with persistent HH.^3^ This suggests that further genetic aetiologies remain to be discovered.

HH has also been reported as a feature in at least 20 different rare genetic syndromes.^1^ The most common is Beckwith‐Wiedemann syndrome where HH occurs in approximately 50% of cases.^4^ More rarely, HH has been described in individuals with other overgrowth disorders including Sotos syndrome^5^ and growth delay syndromes such as Kabuki.^6^ HH has also been reported in some cases with congenital disorders of glycosylation and chromosome abnormalities such as Turner syndrome and Patau syndrome.^7^, ^8^, ^9^, ^10^ For patients with syndromic HH, an early genetic diagnosis is important as this will guide medical management and provide information on prognosis and recurrence risk.

Screening of the syndromic genes is not routinely performed as part of the genetic testing strategy for individuals with HH. The targeted analysis of a gene is usually only performed when there is a clinical suspicion of a specific syndrome in an individual and therefore acts to confirm the clinical diagnosis.^11^ Consequently, the prevalence of mutations in these genes in HH is not known. As many patients are referred for genetic testing at diagnosis of HH, it is possible that some individuals with a mutation in a syndromic gene will not have developed additional extra‐pancreatic features at the time of referral for genetic testing. These patients may therefore not receive the most appropriate genetic testing.

In order to assess the frequency of mutations in the known syndromic genes in individuals with HH of unknown genetic cause, we performed a comprehensive analysis of genome sequencing data from 82 affected infants. Screening patients for mutations in the “syndromic” HH genes irrespective of clinical features has the advantage that a genetic diagnosis can precede development of clinical features and guide clinical management, rather than being confirmatory.

## 2 MATERIALS AND METHODS

### 2.1 Patient details

82 infants with HH diagnosed within the first 12 months of life were referred for genetic testing to the Molecular Genetics Laboratory at the Royal Devon and Exeter NHS Foundation Trust. All patients in the cohort had received a biochemical diagnosis of HH in their local centre. All patients had a blood glucose of <2.8 mmol/L (median 1.9 mmol/L) with a concomitant insulin of >15 pmol/L (median 96 pmol/L) and/or a c‐peptide of >300 pmol/L (see Table 1). In all cases, the HH had persisted for greater than 6 months or had required pancreatic resection following a poor response to treatment. In 15 patients, extra‐pancreatic features were present at the time of referral for genetic testing. None of these patients had received a clinical diagnosis consistent with a known syndromic form of HH at the time of study. Mutations in the *ABCC8*,* KCNJ11*,* HADH*,* HNF4A*,* HNF1A*,* GLUD1*,* GCK,* and *SLC16A1* genes had been excluded in all patients using targeted next‐generation sequencing.^11^ The coding regions of the HH candidate genes, *UCP2* and *HK1*, were also screened but no likely pathogenic variants were identified in the cohort.^12^, ^13^, ^14^

**Table 1:** Clinical characteristics of the 82 patients included in this study. All patients had received a clinical diagnosis of HH from their referring clinician, with biochemical testing undertaken in their local laboratories. When applicable, median values are given with the range

Reported consanguineous	18%
Sex (% male)	48%
Birth weight kg (gestation)	3.5 (39 wk) [2.0‐5.2 (35‐41 wk)]
Current age (y)	6.5 [1‐35]
Age at diagnosis of HH (wk)	4 [0‐48]
Blood glucose at diagnosis (mmol/L)	1.9 [<1‐2.8]
Insulin at time of hypoglycaemia (pmol/L)	96 [15‐365]
C‐peptide at diagnosis (pmol/L)	775 [150‐2400]
Extra‐pancreatic features	n = 15

The study was approved by the North Wales Research Ethics Committee. Consent was obtained from each patient after full explanation of the purpose and nature of all procedures used.

### 2.2 Gene panel and variant calling

We utilized the Phenomizer browser to identify disease entries annotated for hyperinsulinaemic hypoglycaemia (HPO id: 0000825).^15^ A survey of the literature was also undertaken to identify further genes in which mutations have been reported to cause HH as part of a syndrome.

Whole‐genome sequencing was performed on DNA extracted from peripheral blood leucocytes of the 82 probands. All samples were sequenced on an Illumina HiSeq 2500 or Illumina X10 (Illumina, San Diego, California, USA) with a mean read depth of 33.25 (SD 4.25). The sequencing data were analysed using an approach based on the GATK best practice guidelines.^16^ This involved aligning the reads to the hg19/GRCh37 human reference genome with BWA mem, applying Picard for duplicates removal, and GATK IndelRealigner for local realignment and running the base quality score realignment. GATK haplotypeCaller was used to identify variants, which were annotated using Alamut Batch version 1.8 (Interactive Biosoftware, Rouen, France), and variants which failed the QD2 VCF filter or had <5 reads supporting the variant allele were excluded. Variants that passed filtering were confirmed by Sanger sequencing and tested in the parents (details of primer sequences are available on request). CNVs were called by SavvyCNV, which uses read depth to judge copy number states.^17^ Uniparental isodisomy was detected using a hidden Markov model to detect regions with a significant number of variants that were opposite homozygous between the proband and one parent.

## 3 RESULTS

We identified 20 genetic syndromes in which HH has been reported as a feature (Table 2). In 82 patients, we screened the coding regions and intron/exon boundaries of the 18 genes associated with these syndromes. We also searched for copy number variations (CNVs) and evidence of uniparental isodisomy at genomic regions associated with Beckwith‐Wiedemann, Patau and Turner syndromes.

**Table 2:** Syndromes in which hyperinsulinaemic hypoglycaemia (HH) has been reported as a feature. The 18 genes in which mutations have been reported to cause syndromic HH plus the three genomic regions known to be affected by copy number variants (CNVs) or uniparental isodisomy (UPD) are provided

Syndrome	OMIM Gene(s)	Inheritance	References	HH clinical features[Link]
Adenosine kinase deficiency	*ADK*	Recessive	Staufner et al^23^	Neonatal onset. Recurrent. Diazoxide responsive
Congenital disorders of glycosylation (type 1d)	*ALG3*	Recessive	Sun et al^7^	Neonatal onset
Timothy	*CACNA1C*	Dominant	Splawski et al^24^	Childhood onset. Recurrent
*CACNA1D*	*CACNA1D*	Dominant	Flanagan et al^25^	Onset from birth. Persistent and transient reported. Diazoxide responsive
Beckwith‐Wiedemann	*CDKN1C*, UPD or CNVs at 11p15	Dominant	Munns and Batch^4^	Onset in neonatal period. Transient. Diazoxide responsive
Perlman	*DIS3L2*	Recessive	Henneveld et al^26^	Onset from birth
Tyrosinaemia type I	*FAH*	Recessive	Baumann et al^27^	Neonatal onset. Transient. Diazoxide responsive
Simpson‐Golabi‐Behmel	*GPC3*	X‐linked recessive	Terespolsky et al^28^	Neonatal onset
Costello	*HRAS*	Dominant	Sheffield et al^29^	Onset from birth. Transient
Insulin resistance syndrome (leprechaunism)	*INSR*	Dominant	Hojlund et al^30^	Onset 3 to 30 years of age. Postprandial HH. Octreotide responsive
Kabuki	*KMT2D, KDM6A*	Dominant	Gole et al^6^	Onset from birth. Persistent. Diazoxide responsive
Congenital disorder of glycosylation (type 1b)	*MPI*	Recessive	Deeb and Amoodi^31^	Neonatal onset. Persistent. Diazoxide responsive
Sotos	*NSD1*	Dominant	Baujat et al^5^	Neonatal onset. Persistent. Diazoxide responsive
Congenital disorder of glycosylation (type 1t)	*PGM1*	Recessive	Tegtmeyer et al^8^	Childhood onset. Recurrent
Central hypoventilation syndrome	*PHOX2B *	Dominant	Hennewig et al^32^	Neonatal onset. Recurrent. Diazoxide responsive
Congenital disorder of glycosylation (type 1a)	*PMM2*	Recessive	Bohles et al^33^	Neonatal onset. Persistent. Diazoxide responsive
Polycystic Kidney Disease with HH	*PMM2*	Recessive	Cabezas et al^9^	Neonatal/childhood onset. Persistent. Diazoxide responsive
TRMT10A	*TRMT10A*	Recessive	Gillis et al^34^	Childhood onset. Persistent. Diazoxide responsive
Patau syndrome	Trisomy 13	* *De novo	Smith and Giacoia^35^	Onset from birth. Transient
Turner	X Chromosome deletions	*De novo*	Alkhayyat et al^10^	Neonatal onset. Persistent. Diazoxide responsive

Features of HH as reported in cases from the published literature.

We identified 70 nonsynonymous (nonsense, frameshift, splice site, missense) variants in the 18 genes. As the incidence of HH in outbred populations is estimated to be between 1 in 27 000 and 1 in 50 000,^18^ we excluded all variants in dominant genes that were present in gnomAD controls^19^ at a frequency greater than 1 in 27 000. In addition, we excluded variants that did not fit the known inheritance pattern of the syndrome: excluding single heterozygous variants in recessive genes and variants inherited from an unaffected parent in dominant genes. This left one *de novo* variant in the *KMT2D* gene where dominantly inherited loss‐of‐function mutations are reported to cause Kabuki syndrome.^6^

CNVs were not detected at any of the regions analysed, and analysis of single polymorphisms excluded uniparental isodisomy at the Beckwith‐Wiedemann syndrome locus at chromosome 11p15.5. All patients had normal dosage of chromosome 13, and all females had a 46XX karyotype, which excluded Patau and Turner syndromes, respectively.

**Figure 1:**
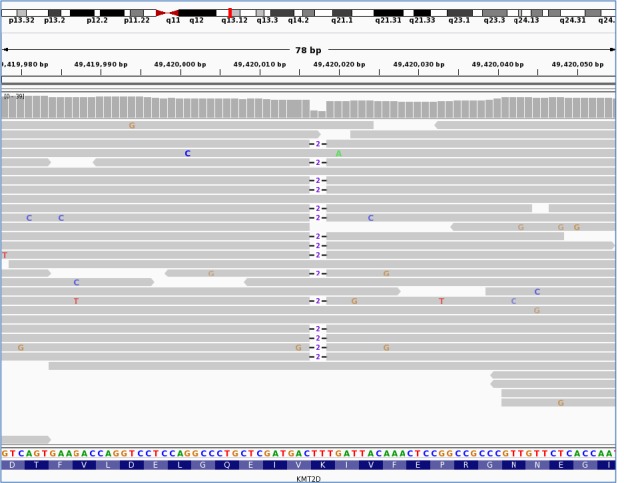
*KMT2D* variant hg19/GRCh37:g.49420017_49420018del/NM_003482:c.15731_15732del/p.Lys5244Serfs*13. Visualized in integrative genomics viewer (IGV). It shows the sequencing reads. (horizontal grey bars) mapping to exon 48 of the *KMT2D* gene located at genomic position 49,420,017 on chromosome 12. The reference nucleotide sequence and the amino acid translation are provided under the sequencing reads. The heterozygous deletion of TT is illustrated by ‐2‐ and is present in 15 of the 25 sequencing reads present at this position. The deletion causes a frameshift.

The heterozygous frameshift variant in the *KMT2D* gene p.(Lys5244Serfs*13), (c.15731_15732del) and (NM_003482.3) (Figure 1) had arisen *de novo* in the proband and was classified as “pathogenic” according to the American College of Medical Genetic guidelines.^20^ The female patient was of Sarawakian ethnicity born in Malaysia to nonconsanguineous parents at 38 weeks of gestation with a birthweight of 3.36 kg. During the pregnancy, her mother was diagnosed with gestational diabetes that was controlled by diet and the pregnancy was complicated by polyhydramnios. Hypoglycaemia was diagnosed in the proband at birth (2.0 mmol/L), which required intermittent intravenous dextrose for recurrent symptomatic hypoglycaemia until day 30 of life. Diazoxide treatment (5 mg/kg/d) and hydrochlorothiazide treatment (1.5 mg/kg/twice daily) were started at 30 days, which resulted in euglycaemia. Hyperinsulinism as the cause of hypoglycaemia was confirmed biochemically. The patient required continuous diazoxide treatment and a nasogastric tube for feeding. At the age of 6 months, the patient was referred for genetic testing of the genes causing isolated HH. At referral, the only extra‐pancreatic feature reported to the genetics laboratory was gastro‐oesophageal reflux. At follow‐up subsequent to the genetic diagnosis, clinical features consistent with a diagnosis of Kabuki syndrome were reported. At the age of 5 months, her weight was 6.06 kg (10th‐50th centile), height was 60 cm (<10th centile), and head circumference was 38.5 cm (<10th centile). She had soft facial dysmorphism, hypotonia and laryngomalacia with inspiratory stridor. Her gross and fine motor development was delayed. She also had a resolved small muscular ventricular septal defect and congenital small right kidney with normal left kidney. She had recurrent pneumonia and died before reaching 1 year of age.

## 4 DISCUSSION

We screened for genetic aetiologies where HH has been reported to feature as part of a complex syndrome in 82 patients with HH of unknown cause. We identified one patient with a pathogenic variant in *KMT2D* that confirmed a diagnosis of Kabuki syndrome.^6^ At referral, the patient was reported to the genetics laboratory as having persistent HH and gastro‐oesophageal reflux. At follow‐up subsequent to the genetic diagnosis, clinical features consistent with a diagnosis of Kabuki syndrome were reported. These included a structural heart defect with growth and developmental delay. It is possible that if the patient had been referred to a specialist clinical geneticist, a clinical diagnosis of Kabuki syndrome would have been made. As the patient died before the age of 1 year, it is not possible to ascertain whether they would have developed further features of this syndrome. Recently, Yap et al^21^ described a cohort of 10 patients with pathogenic variants in *KDM6A* who presented with HH before receiving a diagnosis of Kabuki syndrome later in life suggesting that HH may be a more common presenting feature than previously recognized. Their screening of an additional 100 isolated HH patients identified one further patient (who was retrospectively recognized to have the classic syndromic phenotype), which is a similar frequency to our study.

Mutations in the “syndromic” HH genes were not identified in the majority of our cohort (81/82 patients). Our patients were selected due to the presence of persistent HH rather than a syndromic phenotype. Our low pickup rate suggests that mutations in these genes are rare in patients presenting with isolated HH. It is likely that for the majority of cases with syndromic disease, additional features are evident from birth allowing for a clinical diagnosis followed by confirmatory genetic testing. This would consequently reduce the prevalence of cases within our cohort as the majority of patients are referred for routine screening of the 8 known isolated HH genes. Heterozygous variants in genes that cause a dominant disease were excluded if they were inherited from an unaffected parent; however, it is possible that these could be pathogenic but displaying incomplete penetrance in the parents. We can also not rule out the possibility that some patients have mutations in other genes known to cause multisystem disease which was not screened in this study as HH has not been recognized as a common feature. Furthermore, it is possible that some patients with Beckwith‐Wiedemann syndrome have a methylation defect in the absence of a structural abnormality that was not detected by our analysis.

The finding that the patient with Kabuki syndrome had HH as the presenting feature highlights the potential benefit of screening for the syndromic genes in individuals newly diagnosed with HH. Identifying the underlying genetic aetiology is important for these patients as it will inform on prognosis which will allow for better clinical management. A genetic diagnosis also provides important information on recurrence risk.

We found that mutations causing these 20 syndromes are rare. The recent adoption of targeted next‐generation sequencing by molecular genetics laboratories makes screening of multiple genes for conditions such as HH feasible. An early and accurate genetic diagnosis of a syndrome has benefits for patient care. However, there are some disadvantages of this approach in terms of the difficulties with variant interpretation in the absence of additional clinical features and the extra time required for the analysis of the genetic data. Thus, routine testing of this panel of genes may not be justified for patients without syndromic features as we have shown that mutations in these genes are rare in such a cohort. It is anticipated that variant interpretation will become easier through data sharing initiatives such as ClinVar,^22^ and as the number of publically available control data sets increase, more variants can be excluded by frequency.^19^ This may make testing of these genes in all patients with HH more feasible in future.

Screening large cohorts for mutations in these genes will provide further information on the prevalence of mutations in HH, which will help to guide future genetic screening strategies for this condition and may also identify individuals with “nonclassical” features, which would expand the phenotype associated with these syndromes.

In conclusion, while the clinical impact of finding a mutation in a syndromic gene is high as it can inform clinical management, mutations in the syndromic HH genes are rare in individuals referred for routine testing for HH. Given the time‐consuming nature of a comprehensive screen, routine testing of these genes by molecular genetics laboratories is unlikely to be justified in patients without syndromic phenotypes.

## CONFLICT OF INTEREST

The authors have no conflict of interests to declare.

## AUTHOR CONTRIBUTIONS

TWL and SEF designed the study. JHYH and KH recruited patients and performed clinical phenotyping. TWL and MNW analysed the sequencing data. JALH, SE and SEF performed variant interpretation. TWL and SEF wrote the manuscript. All authors reviewed and approved the manuscript.
